# Abdominal Obesity as a Predictive Factor of Nonalcoholic Fatty Liver Disease Assessed by Ultrasonography and Transient Elastography in Polycystic Ovary Syndrome and Healthy Women

**DOI:** 10.1155/2019/9047324

**Published:** 2019-08-04

**Authors:** Siriluk Tantanavipas, Orawin Vallibhakara, Abhasnee Sobhonslidsuk, Sith Phongkitkarun, Sakda Arj-Ong Vallibhakara, Kwannapa Promson, Areepan Sophonsritsuk

**Affiliations:** ^1^Reproductive Endocrinology and Infertility Unit, Department of Obstetrics and Gynaecology, Faculty of Medicine Ramathibodi Hospital, Mahidol University, Bangkok 10400, Thailand; ^2^Division of Gastroenterology and Hepatology, Department of Medicine, Faculty of Medicine Ramathibodi Hospital, Mahidol University, Bangkok 10400, Thailand; ^3^Department of Diagnostic and Therapeutic Radiology, Faculty of Medicine Ramathibodi Hospital, Mahidol University, Bangkok 10400, Thailand; ^4^Section for Clinical Epidemiology and Biostatistic, Faculty of Medicine Ramathibodi Hospital, Mahidol University, Bangkok 10400, Thailand

## Abstract

Polycystic ovary syndrome (PCOS) and nonalcoholic fatty liver (NAFLD) share similar clinical presentations including obesity, insulin resistance (IR), and metabolic abnormality. The predictive factors of NAFLD in women with PCOS and specifically in Asian women are not well established. Associated factors for NAFLD assessed by ultrasound (US) among a group of PCOS and healthy women were determined and diagnostic accuracy between US and transient elastography (TE) for NAFLD was compared and correlated. Sixty-three women with ages ranging from 20 to 40 years participated in the present cross-sectional study. Forty-two women with PCOS as diagnosed by the Rotterdam criteria and 21 healthy women were recruited into the study. Women with underlying hepatic diseases and history of alcohol consumption >20 g/day were excluded. Biochemical and hormonal testing, anthropometrics, liver US, and TE were assessed. Waist circumference (WC) greater than 80 cm was the only predictive factor for NAFLD as assessed by US in the whole group (adjusted odds ratio [aOR] 5.49, 95% confidence interval [CI]: 1.85–16.26,* p *<0.001). The value of the TE-based controlled attenuation parameter (CAP) was significantly correlated with stage of steatosis as assessed by US (correlation coefficient = 0.696,* p *<0.001). The diagnostic accuracies of dichotomized CAP ≥236 dB/m assessed for NAFLD using US as the gold standard were 84% and 78% sensitivity and specificity, respectively, with the area under the curve at 0.81 (*p *<0.001). Abdominal obesity, rather than the presence of PCOS, was shown to be the independently associated factor for NAFLD. WC could be used as the primary screening tool before performing complicated intervention for detection of steatosis. TE is an alternative noninvasive detection tool in women with PCOS for NAFLD and hepatic fibrosis identification.

## 1. Introduction

Polycystic ovary syndrome (PCOS) is the most common gynecological endocrine disorder. The prevalence of PCOS diagnosed by Rotterdam criteria has been reported approximately 5%–10% in women of reproductive age. PCOS affects women's health with long-term consequences including endometrial hyperplasia, endometrial cancer, and an increase in cardiovascular risk contributed from insulin resistance (IR), type 2 diabetes mellitus, hypertension, and metabolic syndrome (MetS).

Nonalcoholic fatty liver disease (NAFLD) or steatosis refers to an increase in and excessive hepatic fat accumulation. It is identified by the presence of fat >5% of hepatocyte and/or proton density fat fraction technique or >5.6% defined by magnetic resonance spectroscopy. The exclusion of the secondary causes of fatty liver disease and a daily alcohol consumption ≥20 g is required for NAFLD diagnosis [1]. NAFLD is classified as nonalcoholic fatty liver (NAFL) and nonalcoholic steatohepatitis (NASH), which consists of a more progressive and wider clinical spectrum including fibrosis, cirrhosis, and hepatocellular carcinoma [2]. Within five to seven years, NASH can become cirrhosis in almost 20% of the cases [3]; therefore, patients with NASH need closer follow-ups and intensive treatment to prevent NASH progression to cirrhosis and carcinoma. Although NAFLD pathogenesis is multifactorial, obesity and IR are the main contributing factors [4]. Liver biopsy is the gold standard for NAFLD diagnosis [5]. However, this technique is invasive, and it is burdensome to perform liver biopsies in all NAFLD patients. The international guidelines recommend liver biopsy in selected cases consisting of those who may have advanced fibrosis indicated by serum biomarkers or transient elastography (TE) [6]. Ultrasound (US) is a noninvasive technique and initial diagnostic test for assessment of fatty liver. It is good for moderate to severe steatosis but less accurate when fat accumulation is <20% [2] and depends on operator expertise [7]. A parameter assessed by fibroscan or TE, called controlled attenuation parameter (CAP), which measures attenuation of the US at the center frequency of the fibroscan probe, is the other promising tool for steatosis assessment because of its accuracy and convenience [7]. However, results are inconclusive for TE-based CAP performance compared to US for steatosis detection. Additionally, TE is a new and promising technique for liver fibrosis evaluation in order to avoid invasive liver biopsy.

Both PCOS and NAFLD share similar characteristics, including central obesity, IR, and MetS. Many studies have demonstrated an increase in prevalence of NAFLD in women not only with obese but also with nonobese PCOS [8–12]. It is still a question of whether PCOS is one of the predictive factors of NAFLD and specifically in Asian women. Moreover, no recommendations regarding assessment tools for NAFLD in women with PCOS have been published. The present study aimed to examine two parameters: (1) identify the predictive factors of NAFLD by US in PCOS and healthy women and (2) correlate and compare diagnostic accuracy for NAFLD between US and TE-based CAP.

## 2. Material and Methods

Forty-two women with PCOS based on the Rotterdam criteria [13] and twenty-one healthy women with ages ranging from 20 to 40 years provided informed consent and were recruited into the study. The present cross-sectional study was performed at the Endocrine Clinic, Division of Reproductive Endocrinology & Infertility, Department of Obstetrics and Gynecology, Faculty of Medicine Ramathibodi Hospital, Mahidol University, Bangkok, Thailand, between November 2017 and August 2018. The study was approved by the Ethical Clearance Committee on Human Rights Related to Researches Involving Human Subjects, Faculty of Medicine, Ramathibodi Hospital. All participants were excluded if they had other related diseases, such as thyroid dysfunction, hyperprolactinemia, androgen-producing tumor, and Cushing's syndrome in addition to others. The other exclusion criteria included alcohol consumption >20 g/day, presence of known liver disease, and use of steatogenic medication or hormones for at least three months before the study. Medical history recording, physical examination, pelvic and liver US, laboratory testing, and TE were done for the entire group.

*Definition. *Abdominal obesity was defined by WC >80 cm [14]. Hyperandrogenism (HA) was characterized by clinical presentations (such as hirsutism, moderate to severe acne, androgenic alopecia, and seborrhea) [13] and/or biochemical testing. Biochemical HA was defined by a free androgen index (FAI) of >6.8 [15]. MetS was defined by the presence of ≥3 risk factors: (1) WC >80 cm; (2) hypertension >130/85 mmHg; (3) fasting plasma glucose (FPG) >6.1 mmol/L; (4) triglycerides (TG) >1.7 mmol/L; and (5) high-density lipoprotein (HDL-C) <1.3 mmol/L [16]. 

*Anthropometric Measurement. *Women's weights, heights, and WCs women were measured. Body mass index (BMI) was calculated as body weight (kg) divided by the height (m) squared. WC was measured around the body midway between the lower rib margin and the iliac crest by placing measuring tape at that location [14]. A BMI of 23.0 to 24.9 and ≥25.0 kg/m^2^ was interpreted as overweight and obesity, respectively [17]. 

*Laboratory Assays. *All of the blood samples were analyzed for aspartate aminotransferase (AST), alanine aminotransferase (ALT), *γ*-glutamyltransaminase (*γ*-GT), alkaline phosphatase (ALP), albumin (ALB), total cholesterol (TC), TG, HDL-C, low-density lipoprotein cholesterol (LDL-C), insulin, prolactin, total testosterone (T), sex hormone-binding globulin (SHBG), thyroid-stimulating hormone (TSH), follicle-stimulating hormone (FSH), hepatitis B surface antigen (HBsAg), and antihepatitis C virus (anti-HCV). The oral 75 gm-glucose test was done. The FAI was calculated with the formula: TC (nmol/L) x 100 /SHBG (nmol/L). The homeostasis model assessment of IR (HOMA-IR) method was used for IR evaluation when this level >2.77 [18]. HOMA-IR was calculated with the formula: HOMA-IR = insulin (mIU l − 1) × glucose (mmol l − 1)/22.5. 

*Liver Ultrasonography. *US was performed by a radiologist (SP) with gastrointestinal (GI) system-based expertise. Diagnosis according to this method measured the extent of brightness or diffusely increased echogenicity of the liver parenchyma, echogenic discrepancy of the liver and the kidney, and loss echogenicity of portal venous walls [19]. Hepatic steatosis severity was divided into several categories: (1) mild steatosis or steatosis grade 1 (S1) as defined by the presence of bright echoes or increased hepatorenal contrast; (2) moderate steatosis or S2 as defined by both of the presence of bright echoes or increased hepatorenal contrast, and severe steatosis; or (3) S3 included the same criteria as S2 in addition to the presence of posterior beam attenuation [20]. 

*NAFLD and Liver Stiffness Measurement Using Transient Elastography. *After overnight fasting, all participants had TE (Fibroscan®, Echosen, Paris) by a single well-trained nurse (KS). Either a medium (M) or extra-large (XL) probe according to the recommendation of software was placed perpendicular to the skin through an intercostal space on the liver's right lobe while the patient remained in the dorsal decubitus position with his/her right arm maximally abducted. Low-frequency vibrations were then transmitted to the skin and induced a shear wave that propagated through the liver in a spherical manner. The TE measurement was defined as a successful examination when ≥10 validated measurements with an interquartile range (IQR)/median ratio were ≤0.30 [21]. Liver stiffness measurement (LSM) was measured concurrently with CAP. NAFLD stages 1–3 were defined if CAP ≥ 236, 270, and 302 dB/m, respectively [22]. Significant and advanced fibroses and cirrhosis were defined by LSM >7.0, 9.5, and 13.0 kPa, respectively [23–25]. 

*Statistical Analysis. *The demographic data was analyzed using the Student T- and chi-squared tests for normally distributed continuous and discrete data, respectively. The Mann-Whitney U test was used for statistical comparison of nonnormal distributed continuous data. Univariate logistic regression was performed for the entire population and variables with a P-value ≤ 0.15 in the univariate analyses (i.e., WC, BMI, FBS, biochemical HA, IR, abnormal glucose, TG, HDL, LDL, and metabolic syndrome) were entered into the multivariable analyses. A forward stepwise regression with a P-value < 0.05 was used to develop the final multivariable models. Adjusted odds ratio (OR) with 95% confidence interval (CI) for the logistic regression was calculated. The correlations between the CAP values and hepatic steatosis grades assessed by US were analyzed by the Spearman's correlation coefficient and divided into several categories: (1) 0.00 to 0.25 none or slight; (2) 0.25 to 0.50 fair to moderate; (3) 0.50 to 0.75 moderate to good; and (4) 0.75 to 1.00 almost perfect [26]. The receiver-operating characteristic (ROC) curve with area under the curve (AUC) value was measured in order to evaluate the diagnostic accuracy of dichotomized CAP (dCAP) compared to the US (gold standard). The sensitivity, specificity, and positive and negative predictive values were calculated. The dCAP value was categorized as <236 and ≥236 dB/m. A probability value <0.05 (P <0.05) was considered statistically significant. All statistical analyses were performed by Stata, version 15.0 (StataCorp LLC, College Station, TX).

## 3. Results

Clinical and biochemical characteristics of PCOS and healthy women and those of women with and without NAFLD are shown in Tables 1 and 2, respectively. The mean ages of women were not significantly different between women with (n=31) and without NAFLD (n=32) (28.87 ± 6.05 and 29.01 ± 5.33 years, respectively;* p*=0.924). Among the variables, those demonstrating significant difference between the groups with and without NAFLD were WC (*p*=<0.001), BMI (*p*=<0.001), FBG (*p*= 0.039), 2hr glucose (*p*= 0.004), TG (*p*= 0.001), HDL-C (*p*=0.002), HDL-C (*p*=0.015), and MetS (*p*=0.027). Interestingly, the presence of PCOS, HA, and metabolic parameters for IR was not significantly different between women with and without NAFLD.

Results from a univariate binary logistic regression for the whole group showed that NAFLD was significantly associated with WC >80 cm, BMI ≥25 kg/m^2^, FBS ≥ 100, biochemical HA, IR, abnormal glucose, TG, HDL-C <50 mg/dL, LDL-C >130 mg/dL, and MetS (Table 3). After entering all of these factors into the multivariate logistic regression, only WC (>80 cm) remained significantly associated with the presence of NAFLD (adjusted odds ratio [aOR] 5.49, 95% confidence interval [CI]: 1.85–16.26;* p *<0.001) (Table 4).

**Figure 1:**
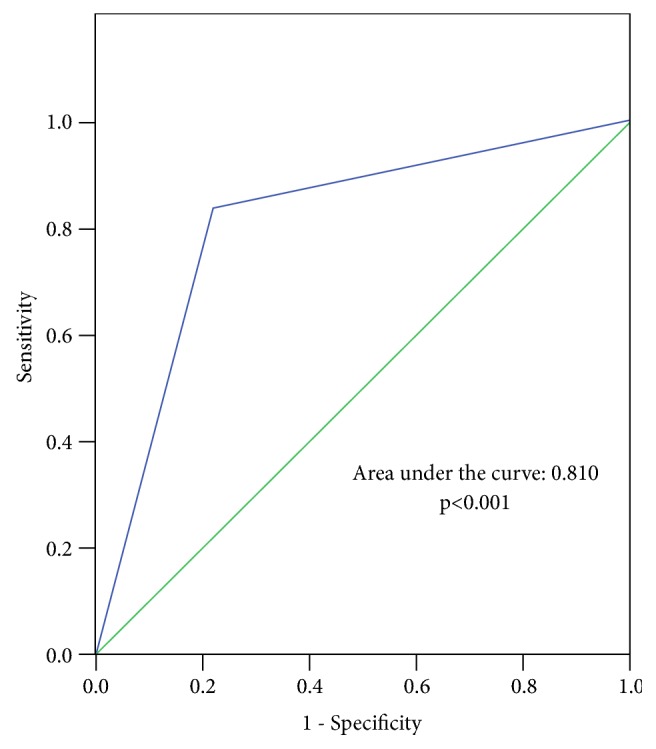
Receiver-operating characteristic curve (ROC) analysis of transient elastography-based controlled attenuation parameter (TE-CAP) for screening NAFLD in patients with PCOS and NAFLD diagnosed by hepatic US. ROC, receiver-operating characteristic curve.

Interestingly, TE-based CAP values significantly correlated with steatosis stage as assessed by US (correlation coefficient 0.696;* p *<0.001). Based on the ROC curve using US as the gold standard, the cut-off value of dCAP at ≥236 dB/m for NAFLD yielded a sensitivity and specificity of 84% and 78%, respectively, with the area under the curve at 0.81 (*p *<0.001) (Figure 1). Steatosis was detected by US in 31/63 (49.21%) and by TE-based CAP in 33/63 (52.38%) (Table 5). Remarkably, NAFLD was detected by TE-based CAP in seven (21.88%) women with normal US findings. On the contrary, it was detected by US in five (16.67%) women with TE-based CAP <236 dB. The discordant rate of the stage of steatosis categorized by US and TE-based CAP was 42.86%. The average BMI and WC of women in the concordant and discordant groups (n=36 and 27, respectively) were significantly different (24.76 ±5.41 kg/cm^2^ and 79.75±12.97 cm and 29.12 ± 6.23 kg/cm^2^ and 89.74 ±13.45 cm, respectively;* p*=0.004 for both BMI and WC).

In addition, two women with PCOS had significant fibrosis as defined by LSM of 7.4 and 7.6 kPa. One of them had steatosis detected by both US and TE-based CAP (291 dB/m) but it was not present in the other one after assessment via both techniques. Both of them were referred to a GI internist, and strict lifestyle modifications were suggested.

## 4. Discussion

NAFLD is manifested by many metabolic factors similar to PCOS, such as MetS, hyperinsulinemia, and IR. Based on the present study, abdominal obesity, as defined by a WC >80 cm, was the predictive factor for NAFLD as assessed by US among both PCOS and healthy women. The correlation and accuracy of TE-based CAP for NAFLD detection were nearly equal to US, but TE provides an advantage over the US for concurrently identifying significant liver fibrosis during CAP assessment.

Abdominal obesity (WC ≥ 80 cm), but neither the total body fat nor the presence of PCOS, was an essential key for the presence of NAFLD in our PCOS and healthy women. The magnitude of OR for the strength of the association of WC and NAFLD from our study was about 5-fold with high precision level. The results of our study parallel those from Petta et al.'s study in PCOS women [27] although the assessment tool for NAFLD was different from ours. We detected NAFLD by US, whereas they defined NAFLD when the hepatic steatosis index was >36. Cerda et al. [28] and Zhang et al. [29] demonstrated the significance of abdominal obesity for the presence of steatosis in women with PCOS by using the waist-to-hip (WHR) ratio, which is the other anthropometric parameter indicating abdominal obesity. Both WC and WHR are the simple anthropometric variables for prediction of the presence of visceral or abdominal fat [14]. Many highly technological techniques, including dual energy X-ray absorptiometry (DEXA), computerized tomography (CT) scan, and magnetic resonance imaging (MRI) have been reported for directly detecting visceral fat/adiposity [30–32]. Previous data have shown that WC correlated with visceral fat deposit as assessed by CT [33]. Moreover, visceral adiposity has been demonstrated for its association with fatty liver in many studies [31–33]. Eguchi et al. and Park et al. showed that visceral fat accumulation as detected by CT-scan correlated with the severity of fatty liver as assessed by US in Japanese subjects and by liver tissue from healthy living donors, respectively [32]. Van der Poorten et al. showed that the visceral fat as evaluated by magnetic resonance imaging (MRI) may be a predictor for hepatic fibrosis [31]. Vassilatou et al. showed a role for the visceral adiposity index (VAI) in NAFLD detection [34]. Additionally, we confirmed the significance of central obesity on NASH development as previously demonstrated in Thai patients shown in Sobhonslidsuk et al.'s study [30]. Additional studies focusing on visceral adiposity in NAFLD using high technology (such as dual energy X-ray absorptiometry [DEXA], computed tomography [CT] scan, or MRI) would be required to directly reveal the pathogenesis of this disease. Visceral adiposity may increase free fatty acid flow to the liver resulting in the accumulation of fat and alterations in insulin sensitivity.

HA and IR were not independent factors for NAFLD in our study. Our results were incompatible with the studies performed by Vassilatou et al. [10], Kim et al. [12], and Cai et al. [35]. Criteria for PCOS diagnosis and the participants' characteristics may have influenced the outcomes. In a study performed by Vassilatou [36] Androgen Excess PCOS (AE-PCOS) criteria were used and demonstrated the predictors as FAI and a decrease in SHBG and HOMA-IR, but the same researchers in 2018 identified VAI as a predictive factor for PCOS diagnosis after using the Rotterdam criteria [34]. Since the AE-PCOS criteria require patient to have either clinical or biochemical HA, the Rotterdam criteria require two out of three parameters (HA and US-based PCOM findings and oligomenorrhea) to diagnosis PCOS in which some patients might not present with HA. Subjects in the NAFLD group of Kim's and Cai's studies had higher blood glucose levels, HOMA scores, FAI, and serum ALT than those in the control group, which is different from our study [12, 35]. These parameters were not significantly different between women with and without NAFLD in our study.

PCOS presence is controversial for its predictive role in NAFLD. We found no significant effects of PCOS on NAFLD, which is consistent with the study performed by Bohdanowicz-Pawlak et al. [37]. The Rotterdam criteria and US were used for PCOS and NAFLD diagnoses, respectively, in both our and their studies. However, in a large longitudinal cohort study conducted in the United Kingdom by Kumarendran et al., it was demonstrated that women with PCOS had an increased rate of NAFLD with a hazard ratio of 2.23. However, several limitations in the Kumarendran study were found, including the incompleteness of data record, unknown criteria for diagnosis of PCOS and NAFLD, and the uncertainty of the recording method for clinical presentation [38].

The present study demonstrated moderate-to-good correlation between TE-based CAP value and steatosis stage as detected by US and high diagnostic accuracy, sensitivity, and specificity of TE-based CAP for detecting NAFLD based on US. US is used and acceptable in the clinic for detecting fatty liver. However, the availability of a radiologist with expertise in GI system and reliability for assessment steatosis if <20% of fat accumulation can be major limitations [39, 40]. TE-based CAP, a new algorithm measuring total US attenuation at the central frequency of the TE probe, strongly correlated with liver fat accumulation in another study [41]. The correlation between TE-based CAP values and stage of steatosis based on US based on the present study was consistent with studies by Yen et al. and Ferraioli et al. [42]. Our results showed good sensitivity and specificity of TE-based CAP compared to US, which was parallel to the recent study done by Ferraioli et al. although they examined pediatric patients and used a CAP cut-off value of 249 dB/m for steatosis diagnosis [43].

A disagreement with respect to steatosis stage as detected by TE-based CAP and US was demonstrated in our study and was related to obesity. US has detection limitations when fat accumulation <20% [39] or elevated BMI (>40 kg/m^2^) [44]. Obesity is not only an obstacle for US but also TE-based CAP for steatosis detection. The increase in skin capsular distance and tissue between skin and liver epidermis results in an error for detecting steatosis based on CAP [45]. TE-based CAP detected many more subjects with steatosis than US based on our study. However, it is not possible to conclude whether TE-based CAP overestimated or US underestimated NAFLD because we lacked liver biopsy (gold standard) results for comparison.

Some limitations were present in our study. We did not perform any liver biopsies, the gold standard for NAFLD diagnosis, but it is unrealistic to do liver biopsies in young women with PCOS because of the method's invasiveness. Moreover, sampling error and requirements for highly trained physicians and pathologists introduced another disadvantage. However, we minimized the errors as much as possible using US performed by a radiologist with significant experience in GI system following the standard TE protocol for NAFLD diagnosis written by the Echosen company with regular machine inspections and validation.

## 5. Conclusions

Due to the wide-spectrum of the disease from simple steatosis to hepatocellular carcinoma and the currently limited effective treatments, the most important factor for NAFLD management was shown to be early detection and prevention of disease progression. Abdominal obese patients, whether or not they have PCOS, are at risk for NAFLD. TE is an alternative useful and realistic tool for NAFLD detection and concurrent identification of hepatic fibrosis in patients with PCOS.

## Figures and Tables

**Table 1:** Baseline characteristics of polycystic ovary syndrome (PCOS) and healthy women.

Characteristics	PCOS women (n=42)	Healthy women (n=21)	Pvalue
Age (years)	27.71 ± 5.23	31.40 ± 5.79	0.013
WC (cm)	84.81 ± 14.70	82.48 ± 12.64	0.537
BMI (kg/ m^2^)	27.05 ± 6.59	25.79 ± 5.13	0.448
T (ng/mL)	42.36 ± 21.58	30.62 ± 11.32	0.023
SHBG (nmol/L)	36.16 ± 36.67	43.21 ± 24.66	0.043
FAI	8.05 ± 8.17	3.92 ± 4.07	0.009
FBG (mg/dL)	87.17 ± 9.61	84.71 ± 7.94	0.317
2hr Glucose (mg/dL)	112.55 ± 44.62	91.62 ± 25.81	0.052
Insulin (uIU/mL)	20.65 ± 23.35	12.56 ± 10.51	0.136
HOMA-IR	4.51 ± 4.97	2.75 ± 2.51	0.134
AST (U/L)	22.86 ± 7.86	19.52 ± 4.23	0.075
ALT (U/L)	25.62 ± 16.22	23.14 ± 22.06	0.220
*γ*-GT (U/L)	25.02 ± 16.60	20.71 ± 7.85	0.535
ALP (U/L)	68.12 ± 16.97	58.21 ± 18.32	0.615
ALB (g/L)	37.65 ± 2.00	38.07 ± 2.21	0.457
TG (mg/dL)	104.31 ± 71.94	84.71 ± 36.59	0.246
TC (mg/dL)	199.0 ± 38.67	205.48 ± 34.53	0.519
HDL-C (mg/dL)	50.76 ± 13.69	50.86 ± 10.88	0.978
LDL-C (mg/dL)	134.00 ± 35.47	141.05 ± 32.06	0.446
TSH (uIU/mL)	1.83 ± 0.93	1.70 ± 0.93	0.607
PRL (ng/mL)	17.13 ± 9.54	23.54 ± 17.37	0.127
FSH (mIU/mL)	4.11 ± 1.51	4.06 ± 1.53	0.898
Metabolic syndrome (n,%)	6 (14.29)	3 (14.29)	1.000

Note: WC, waist circumference; BMI, body mass index; T, total testosterone; SHBG, sex hormone-binding globulin; FAI, free androgen index; FBG, fasting blood glucose; HOMA-IR, homeostatic model assessment of insulin resistance; AST, aspartate; ALT, alanine aminotransferase; *γ*-GT, *γ*-glutamyltransaminase; ALP, alkaline phosphatase; ALB, albumin; TG, total cholesterol; TC, triglyceride; HDL-C, high-density lipoprotein cholesterol; LDL-C, low-density lipoprotein cholesterol; TSH, thyroid-stimulating hormone; PRL, prolactin; FSH, follicle-stimulating hormone; PCOS, polycystic ovary syndrome. P<0.05 was considered statistically significant. Values are expressed as mean±SD or number, percentage (n, %).

**Table 2:** Baseline characteristics of women with and without NAFLD assessed by abdominal ultrasonography (US).

Characteristics	NAFLD (n=31)	No NAFLD (n=32)	P-value
Age (years)	28.87 ± 6.05	29.01 ± 5.33	0.924
WC (cm)	91.19 ± 13.89	77.09 ± 10.18	<0.001
BMI (kg/ m^2^)	29.86 ± 6.06	23.49 ± 4.35	<0.001
T (ng/mL)	37.71 ± 18.46	39.16 ± 20.73	0.771
SHBG (nmol/L)	34.34 ± 40.78	42.55 ± 23.44	0.329
FAI	8.42 ± 8.83	4.98 ± 5.02	0.061
FBG (mg/dL)	88.74 ± 10.06	84.03 ± 7.51	0.039
2hr Glucose (mg/dL)	120.19 ± 46.83	91.41 ± 27.02	0.004
Insulin (uIU/mL)	19.96 ± 11.99	16.02 ± 25.98	0.445
HOMA-IR	4.49 ± 2.91	3.37 ± 5.41	0.315
AST (U/L)	21.55 ± 6.45	21.94 ± 7.61	0.828
ALT (U/L)	25.45 ± 13.21	24.16 ± 22.25	0.781
*γ*-GT (U/L)	24.90 ± 9.60	22.31 ± 17.89	0.479
ALP (U/L)	65.87 ± 15.49	63.79 ± 20.18	0.649
ALB (g/L)	37.31 ± 2.02	38.26 ± 2.03	0.067
TG (mg/dL)	124.45 ± 76.09	71.94 ± 29.65	0.001
TC (mg/dL)	209.06 ± 35.14	193.50 ± 38.05	0.097
HDL-C (mg/dL)	45.81 ± 12.13	55.63 ± 11.53	0.002
LDL-C (mg/dL)	146.87 ± 35.47	126.16 ± 30.25	0.015
TSH (uIU/mL)	2.01 ± 1.04	1.57 ± 0.75	0.056
PRL (ng/mL)	21.29 ± 15.03	17.31 ± 10.34	0.223
FSH (mIU/mL)	3.96 ± 1.69	4.22 ± 1.31	0.492
PCOS (n,%)	22 (71.00)	20 (62.50)	0.476
Metabolic syndrome (n,%)	8 (25.81)	1 (3.13)	0.027

Note: WC, waist circumference; BMI, body mass index; T, total testosterone; SHBG, sex hormone-binding globulin; FAI, free androgen index; FBG, fasting blood glucose; HOMA-IR, homeostatic model assessment of insulin resistance; AST, aspartate; ALT, alanine aminotransferase; *γ*-GT, *γ*-glutamyltransaminase; ALP, alkaline phosphatase; ALB, albumin; TG, total cholesterol; TC, triglyceride; HDL-C, high-density lipoprotein cholesterol; LDL-C, low-density lipoprotein cholesterol; TSH, thyroid-stimulating hormone; PRL, prolactin; FSH, follicle-stimulating hormone; PCOS, polycystic ovary syndrome. P <0.05 was considered statistically significant. Values are expressed as mean±SD or number, percentage (n, %).

**Table 3:** Univariate analysis of various factors for NAFLD assessed by abdominal US in women with and without NAFLD.

Factors	No NAFLD n(%)	NAFLD n(%)	Odd ratios	95 %CI	P-value
WC					
≤ 80	21(72.41)	8(27.59)	1	-	0.002
> 80	11(32.35)	23(67.65)	5.49	1.66- 18.11	
BMI					
< 25	20(68.97)	9(31.03)	1	-	0.008
≥ 25	12(35.29)	22(64.71)	4.07	1.32- 12.60	
FBS					
< 100	31(54.39)	26(45.61)	1	-	0.104
≥ 100	1(16.67)	5(83.33)	5.96	0.61- 58.51	
2 hrs Glucose					
< 140	29(52.73)	26(47.27)	1	-	0.474
≥ 140	3(37.50)	5(62.50)	1.86	0.40-8.73	
Biochemical HA					
No	25(59.52)	17(40.48)	1	-	0.064
Yes	7(33.33)	14(66.67)	2.94	0.94-9.20	
Clinical HA					
No	19(59.38)	13(40.63)	1	-	0.166
Yes	13(41.94)	18(58.06)	2.02	0.72-5.65	
Total HA					
No	16(64.00)	9(36.00)	1	-	0.123
Yes	16(42.11)	22(57.89)	2.44	0.83-7.15	
Insulin Resistance(IR)					
No	21(63.64)	12(36.36)	1	-	0.045
Yes	11(36.67)	19(63.33)	3.02	1.03-8.85	
Abnormal Glucose					
No	28(60.87)	18(39.13)	1	-	0.015
IGT	4(28.57)	10(71.43)	3.89	0.99-15.23	
DM	0(0.00)	3(100.00)	4.55	0.48-43.07	
TG					
< 150	31(55.36)	25(44.64)	1	-	0.053
≥ 150	1(14.29)	6(85.71)	7.44	0.76-72.47	
HDL					
< 50	10(31.25)	22(68.75)	5.38	1.65-17.56	0.002
≥ 50	22(70.97)	9(29.03)	1	-	
LDL					
≤ 130	19(63.33)	11(36.67)	1	-	0.058
> 130	13(39.39)	20(60.61)	2.66	0.92-7.66	
Metabolic Syndrome					
No	31(57.41)	23(42.59)	1	-	0.013
Yes	1(11.11)	8(88.89)	10.78	1.09-106.31	
PCOS					0.479
No	12(57.14)	9(42.86)	1	-	
Yes	20(47.62)	22(52.38)	1.47	0.50-4.27	

**Table 4:** Multivariate logistic regression analysis of risk factors for NAFLD as assessed by abdominal US in women with and without NAFLD.

	Univariate			Multivariate		
	OR	95% CI	P-value	aOR	95% CI	P-value
WC (cm)						
>80	5.49	1.66–18.11	0.002	5.49	1.85-16.26	<0.001
≤80						

Note: WC: waist circumference, HDL-C: high-density lipoprotein cholesterol, BMI: body mass index, OR: odds ratio, aOR: adjusted odds ratio, and 95% CI: 95% confidence interval.

**Table 5:** Stage of NAFLD detected by US and TE in PCOS and healthy women.

	Detected by US (N, % of total) (n=31)				
Detected by TE (N, % of total) (n=33)	No steatosis	Steatosis grade 1	Steatosis grade 2	Steatosis grade 3	Total (n=63)
No	25	5	0	0	30
NAFLD	(39.68)	(7.94)	(0)	(0)	(47.62)
NAFLD	5	8	0	0	13
Stage 1	(7.94)	(12.70)	(0)	(0)	(20.63)
NAFLD	1	4	1	1	7
Stage 2	(1.59)	(6.35)	(1.59)	(1.59)	(11.11)
NAFLD	1	3	7	2	13
Stage 3	(1.59)	(4.76)	(11.11)	(3.17)	(20.63)

Total	32	20	8	3	63
(n=63)	(50.79)	(31.75)	(12.70)	(4.76)	(100)

## Data Availability

The data used to support the findings of this study are available from the corresponding author upon request.
